# OSpRad: an open-source, low-cost, high-sensitivity spectroradiometer

**DOI:** 10.1242/jeb.245416

**Published:** 2023-07-12

**Authors:** Jolyon Troscianko

**Affiliations:** Centre for Ecology and Conservation, University of Exeter, Penryn Campus, Treliever Road, Penryn, Cornwall TR10 9FE, UK

**Keywords:** Spectrometry, Spectroradiometry, ALAN, Artificial light, Light pollution, Light measurement, Visual ecology

## Abstract

Spectroradiometry is a vital tool in a wide range of biological, physical, astronomical and medical fields, yet its cost and accessibility are frequent barriers to use. Research into the effects of artificial light at night (ALAN) further compounds these difficulties with requirements for sensitivity to extremely low light levels across the ultraviolet to human-visible spectrum. Here, I present an open-source spectroradiometry (OSpRad) system that meets these design challenges. The system utilises an affordable miniature spectrometer chip (Hamamatsu C12880MA), combined with an automated shutter and cosine-corrector, microprocessor controller, and graphical user interface ‘app’ that can be used with smartphones or desktop computers. The system has high ultraviolet sensitivity and can measure spectral radiance at 0.001 cd m−2 and irradiance at 0.005 lx, covering the vast majority of real-world night-time light levels. The OSpRad system's low cost and high sensitivity make it well suited to a range of spectrometry and ALAN research.

## Teaser

**Summary:** The OSpRad spectroradiometer uses off-the-shelf components and 3D-printed parts, and can be controlled via smartphone. It operates from approximately 320 to 880 nm, and at low light down to approximately 0.001 cd m^−2^.

## INTRODUCTION

The night-time light environment is characterised by extreme differences in light intensity and spectral composition compared with typical daytime lighting. This is due to temporal and spatial variation in natural light sources and artificial light at night (ALAN), together with highly variable degrees of atmospheric scattering and reflection. ALAN is a widespread and growing source of environmental pollution that is emitted by street lights, homes and businesses that dramatically alters the timing and spectral composition of the night light environment (Gaston et al., 2013; Kyba et al., 2017; Sánchez de Miguel et al., 2022). The detrimental effects of ALAN have been demonstrated in a wide range of taxa and ecosystems (Owens and Lewis, 2018; Sanders et al., 2018), affecting animal behaviour (Becker et al., 2013; Jolkkonen et al., 2023), physiology (Dominoni et al., 2013), pollination (Knop et al., 2017; Macgregor et al., 2019), sexual signalling (Lewis et al., 2020) and population dynamics (van Grunsven et al., 2020). Measuring the spectral properties of the night-time light environment is therefore essential for ALAN research; however, the technological requirements for this are typically beyond the abilities of standard spectroradiometric equipment, and cost is a limit to widespread data collection.

Animal visual systems vary in their sensitivity to different spectral ranges and intensities of light substantially (Kelber et al., 2017). Like most terrestrial vertebrates, humans have retinas with cones that function in typical daytime light intensities, and rods that provide achromatic vision at low light levels. Even among species with these dual function retinas there is considerable variation in the degree of low-light sensitivity, with adaptations such as rod-dominated retinas, powerful optics and reflective layers that boost low-light sensitivity (Land and Nilsson, 2012). However, a growing number of species have been found to possess chromatic low-light vision, and sensitivity into the ultraviolet range (Kelber et al., 2017). This includes frogs and toads that have evolved cone-like rods (Koskelainen et al., 1994; Yovanovich et al., 2017), geckos that possess three classes of rod-like cones (Roth and Kelber, 2004), and nocturnal bees and hawkmoths that have the ability to discriminate colour down to star-light levels of illumination (Kelber et al., 2002; Warrant and Somanathan, 2022). Measuring the spectral composition of the night-time light environment is therefore essential for understanding the potential effects on the vision of different species, and can be a useful tool for predicting how light impacts the visual ecology of various species. For example, artificial light sources with narrow peaks in their spectral emissions could cause unpredictable interactions between light intensity, object reflectance and colour discrimination for hawkmoths (Briolat et al., 2021). The spectral composition of light also affects a number of other key biological processes; for example, shortwave light in particular is used by plants to detect photoperiod, and is used to regulate the circadian rhythms of many species (Bennie et al., 2016; Grubisic et al., 2019; Sánchez de Miguel et al., 2022). Taken together, this demonstrates the need for further research into the effects of ALAN using measurements that capture the ecologically relevant intensity, spectral, spatial and temporal properties of the light environment.

The night-time light environment can be measured using a range of radiometric techniques that are reviewed by Hänel et al. (2018), and I give a brief overview here with a focus on the principles likely to be important to biologists. One-dimensional measurements such as those provided by lux meters are comparatively affordable and straightforward to acquire, yet they lack the spectral information likely to be important for the reasons outlined above. The spectral sensitivity of a lux meter is also often unknown, and unlikely to match the spectral response curves of humans or any other animal well. As such, the measurements are unreliable when measuring light sources that have narrow peaks in their emission spectra (e.g. the exact location of that spectral emission peak relative to the device's spectral sensitivity function will dramatically affect the measurement, either underestimating or overestimating luminance). Low-cost sensors are also typically unable to perform adequately under low-light conditions (e.g. below ∼10 lx). Camera-based techniques are valuable because they provide both spatial and spectral information; when carefully calibrated, such systems can be used to quantify radiance across the entire visual scene (Nilsson and Smolka, 2021). However, the limited spectral range of camera systems means they are not well suited to accurate modelling of animal visual systems or other processes that are spectrum-specific, e.g. widely used calibrated photography methods that convert from camera sensitivities to animal photoreceptor catch require knowledge of the illuminant spectrum (Troscianko and Stevens, 2015), which is highly consistent in typical daytime terrestrial conditions, but under most low-light conditions the illumination spectrum is often composed of unknown contributions of multiple artificial and natural sources. This can cause metameric effects whereby combinations of (potentially wildly) different illuminant spectra can combine with a single reflectance spectrum to create identical camera tristimulus (RGB) values. Therefore, even if the camera's spectral sensitivity functions are known it may be impossible to reliably or accurately infer photoreceptor catch values under real-world ALAN conditions. Requirements for UV sensitivity add further substantial difficulties to camera-based systems owing to the extremely low sensitivity of camera CMOS sensors to UV spectra and the lack of any wide-angle UV-visible apochromatic lenses. Spectroradiometry offers the highest spectral resolution of any method, and can be used to either measure spectral radiance (light intensity in watts or quanta measured from a specified solid angle per unit area) or spectral irradiance (light measured per unit area, typically using a cosine corrector that collects light from a planar surface). Spectroradiometers allow spectral resolution to be sacrificed for higher low-light sensitivity by using larger aperture slits, so systems are typically configured for the anticipated use (high sensitivity or high spectral resolution). Although radiance is only measured in a narrow incident angle depending on configuration, collimating lenses and mechanical controllers can be used to build up spatial information across the night sky (Hänel et al., 2018) or build up a hyper-spectral image. Although spectroradiometry is often considered the gold standard for radiometric measurements, its widespread use by biologists remains limited, and this is particularly true for those wishing to measure ALAN or natural low-light levels across the UV and human-visible portions of the spectrum. The availability, accessibility and cost of commercial systems that meet the typical requirements of ALAN researchers acts as a real impediment to progress in the field. This is particularly true for data collection over large spatial and temporal scales, and in low-income nations where the effects of ALAN could present significant yet unknown risks to biodiversity.

The Hamamatsu C12880MA is a micro spectrometer that is principally designed for integration into medical components. However, its specifications appear well suited for use in biological and ALAN research with a spectral range of ∼320 to 850 nm, which covers the wavelengths relevant to key biological processes. It is also designed to offer good low-light sensitivity with a 288-site CMOS sensor, integrated optics and comparatively large 500 μm slit size. Its typical spectral resolution according to the manufacturer (full width at half maximum, FWHM) of ∼9 nm (maximum of 15 nm) is also well suited to biological applications where a higher spectral resolution is rarely necessary given typical receptor spectral sensitivity curves (Kelber et al., 2003; Warrant and Somanathan, 2022). The unit is commercially available at low cost [around £220 ($275) at the point of writing; Hamamatsu UK]. However, it is only available as a bare electrical component that requires considerable further integration with circuitry that can control the sensor and read the output (off-the-shelf prototyping boards are considerably more expensive than the units themselves). Moreover, in its basic state it is not well suited to automated low-light measurements that require controlling for the sensor's non-linearities and dark response.

**Fig. 1.**
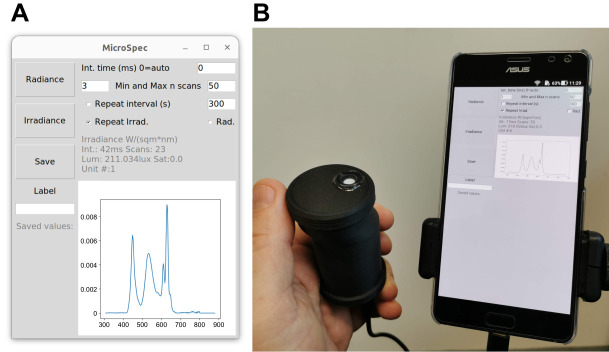
**App with graphical user interface for controlling OSpRad units.** (A) The software running on a Linux desktop, and (B) the same code running on an Android smartphone (connected via a standard USB-C to female USB type A cable).

In this paper, I describe and test an open-source spectroradiometry (‘OSpRad’; Fig. 1) solution for researchers that can meet the design challenges described above at an exceptionally low cost [ca. £250 ($310); see GitHub for parts list]. This utilises 3D printed parts, an open source microcontroller (Fig. S1), automated radiance and irradiance measurement capability (utilising a cosine corrector and shutter), and a user interface capable of running the device from a low-cost smartphone or almost any computer capable of running Python (www.python.org) and connecting via USB. The interface also provides a straightforward repeat timer function for automated data logging, and can readily be modified and integrated with other equipment. This paper details the design and construction (Materials and Methods), and calibration results from five OSpRad devices.

## MATERIALS AND METHODS

### Design

#### Spectrometer controller

The C12880MA chip must be interfaced through a series of input/output electrical pins that control the integration time (the time over which light is collected) and provide output from each of the 288 photosites. Each photosite's peak wavelength sensitivity is described by a function unique to each device, provided by the manufacturer. CMOS photosites increase their voltage with the energy of light they receive; this voltage is then converted to a digital count via an analogue-to-digital converter (ADC). I used an Arduino Nano microcontroller to interface with the C12880MA because they are small, affordable and readily available (the Nano units used here were manufactured by Elegoo, or unbranded manufacturers). The OSpRad unit receives power and communication via the Arduino's USB port, while a serial connection to a desktop computer or smartphone is used to send commands and receive data. I developed the code (‘firmware’) for the Arduino based on the manufacturer's specifications, partially inspired by existing open-source code (see https://github.com/impfs/review). The ADC on the Arduino Nano offers 1024 linear levels, and the sensor's output voltage range limits this to approximately 900 linear levels (this decreases with exposures >∼1 s). This would give the system a maximum theoretical dynamic range of 900:1, which can be improved dramatically by averaging over multiple exposures, and will also be reduced under noisy low-light situations. The controller's default behaviour is to record up to a maximum of 50 exposures, which it then averages at each photosite for each measurement. When 50 exposures would cause the measurement to take longer than 1 s, the code reduces the number of averaged exposures to be less than 1 s in total, down to a minimum of three exposures (although these numbers are all adjustable, Fig. 1). The firmware automatically estimates the ideal integration time by incrementally taking test exposures and doubling the integration time until any part of the spectrum reaches the saturation point. Once saturation is reached, the firmware uses the preceding (unsaturated) measurement to calculate the exposure that would cause the peak intensity measurement to be 0.8 of that which would cause saturation. The maximum exposure is 30 s, above which the system would need additional stability testing (initial testing suggested some photosites may suffer voltage collapse). As with any CMOS sensor, longer exposures increase the noise associated with measurement, and with low light levels the voltage at each site that represents ‘zero’ (the dark noise) is uncertain. Therefore, following each measurement (which will be an average of multiple exposures as described above), the firmware closes a mechanical shutter and takes a dark measurement using an identical exposure regime (the same integration times and numbers of exposures), and uses this to calculate the black point at each site. ADC counts *c* at each photosite *p*, are calculated as:
(1)

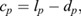
where *l* is the light measurement and *d* is the dark measurement. This controls for temperature and voltage fluctuations that affect the sensor's black point. When exposures are longer than 1 s, the dark measurements are interleaved between light measurements, so that long exposures have closer temporal matching of black point measurements. In addition to this automated exposure behaviour, the user can also manually control the integration time, number of scans and frequency of repeat measures (and whether repeat measures are radiance, irradiance or both).

### Electronics and housing

The housing was created in Blender (version 1.92), and was designed to make device small, robust, easy to print and with the potential for waterproofing (for data logging, see below). All parts here were printed on a Prusa i3 MK3 with ZIRO Matte black PLA filament (amazon.co.uk). I recommend printing in black PLA (polylactic acid) or ABS (acrylonitrile butadiene styrene), and ensuring the shutter blocks near-infrared light [e.g. black PET (polyethylene terephthalate) often transmits near IR, so is not suitable]. The shutter is controlled by a digital servo (Savox SH-0256, www.westlondonmodels.com), which was chosen because of its low cost, availability and relative simplicity. Each servo has a slightly different positional response to the pulse-width-modulated position signal from the Arduino nano. Each OSpRad unit therefore requires the servo's three shutter wheel positions (closed, radiance and irradiance) to be calibrated when first constructed.

### Construction

Construction of this device requires some soldering, access to 3D printed parts (Fig. S1; there are services that offer this through the internet) and a computer that can run the Arduino IDE software (Linux/Windows/MacOS) to write the firmware code to the Arduino nano. Please consult the GitHub construction guide for detailed assembly information (https://github.com/troscianko/OSpRad). The housing was designed to be compact with the potential to make it weatherproof/waterproof for remote logging installations. Waterproofing could be achieved either by painting the 3D-printed housing or printing with ABS plastic and using an acetone vapour bath to fuse the surface. Rubber O-rings are also required for waterproofing the seals (which could be purchased, or made from a sheet of silicone). The user should also select the desired level of protection for the aperture. For purely lab-based measurements, the unit will not require any physical cover for the aperture, and using no cover will improve the performance of the cosine corrector at shallow angles (see Fig. S4). However, I chose to use protective plastic covers (circles of 16 mm diameter, 2 mm thick, made from UV-transmitting sunbed-grade PMMA, Bay Plastics Ltd), cut with a laser cutter. The material can easily be cut into square tiles that would also be suitable. Fused silica microscope cover slips would also function well as protective covers, and would have slightly superior transmission (though would be less impact resistant). The cosine corrector can also be made in different ways. An ideal cosine corrector simply scatters transmitted light in all directions equally, resulting in illuminance of its surface facing the spectrometer that follows a cosine function. I made units that either had a disk of 0.5 mm thick virgin PTFE (Bay Plastics Ltd), sanded on both sides with 180 grit sand paper in a circular motion, or by sandwiching four layers of plumber's PTFE tape (Silverline) in different directions at each layer.

Further optional equipment includes a calibrated light source for more accurate absolute measurements (see below), and an Android smart phone or computer to connect to the spectroradiometer via USB serial interface.

### User interface

I developed a graphical user interface application (‘app’) that can control OSpRad units from either a desktop computer or a smartphone, making the system highly portable and flexible. The app was designed to be easy to use, and suited to both point measures and regular data logging. When a user selects either ‘Radiance’ or ‘Irradiance’ buttons, the app uses the default automated measurement behaviours described above. However, the user also has options for manually controlling the integration time and number of scans to average. Regular repeat measures can be made automatically by ticking the check-box, with a field for inputting the time delay between measurements (the default shown in Fig. 1 is 300 s). Further check-boxes allow the user to specify whether repeat measures should be radiance, irradiance or both. A graph shows the spectrum of the previous measurement [radiance (*L*_e_) or irradiance (*E*_e_) values], together with calculations of luminance (cd m^−2^) or illuminance (lux, lm m^−1^) based on the CIE *y* 2006 analytical function (the app generates the shape of the Y function to match each unit's spectral response). The number of saturated photosites are also shown in the ‘Sat.’ field. Any photosites with saturated values will give underestimates of the flux, and importantly, saturation can only be determined from the light (*l*) measures above (before the dark values are subtracted). As such, any measurements that have multiple saturated values should be treated cautiously and avoided where possible. Clicking the ‘Save’ button will save the current measurement, appending it to the data file. After saving, the app shows a list of recently saved spectra, and prevents re-saving until a new measurement is made.

Data are saved in a manner designed to be both user-friendly and non-destructive. Along with calibrated spectral data [*L*_e_ (W sr^−1^ m^−2^ nm^−1^) or *E*_e_ (W m^−2^ nm^−1^) values at each photosite] and wavelength data for each photosite, the app also saves the unit identifier code (which is used for looking up unit-specific calibration data), the user-specified label, the time and date of the recording, the type of measurement (radiance or irradiance), the integration time and number of scans, the number of saturated values, and the raw spectral count data (*c*). These raw count data have not been calibrated, meaning any measurement can be re-calibrated post-recording. This is potentially useful if a researcher needs to take spectral recordings before they are able to fully calibrate the equipment, or to apply a dark correction (see below). The data are all appended to a ‘data.csv’ file in the same directory as the app's code. The app also requires calibration data to be present in the same directory, stored in a file called ‘calibration_data.csv’. This contains the wavelength, linearisation and radiance/irradiance spectral sensitivity calibration data and coefficients for each unit.

The app was developed in IDLE (version 3.10.6), and written in Python (version 3.10.6), with the graphical user interface based on tkinter (version 8.6.12). The code has a limited number of additional dependencies that need to be installed alongside Python 3: matplotlib, usbserial and (for Android only) usbserial4a. On Android smartphones, the code can be run via the free Pydroid 3 app (see Fig. 1). Adapting the code to support Windows or MacOS will also be straightforward by adding system-specific serial connection details.

### Calibration

Measurements of absolute radiance or irradiance require two main types of calibration: linearisation (ensuring sensor responses scale linearly with flux) and spectral sensitivity (which is a function of optical transmission, diffraction grating efficiency and CMOS sensor sensitivity). The GitHub repository contains all calibration data and calculations for the five units tested here (https://github.com/troscianko/OSpRad). Note that calibration should only be performed once the entire system has been assembled (including the cosine corrector and any protective cover). Units should be recalibrated following any modification to the cosine corrector, protective cover, filter wheel position or major changes to the electronics. Spectrometer calibrations should likely also be repeated after 1 year; however, this will depend on use, e.g. units left outside for data-logging over many days/weeks should be recalibrated more regularly.

#### Linearisation

Although CMOS sensors typically have highly linear relationships between flux and voltage, I found there to be a non-linear response of the system, particularly at low count values (lower counts underestimated the flux), which needs to be controlled for to ensure low-light measurements do not underestimate low-light intensity. Linearity was modelled by measuring the radiance of an AMOLED screen (smartphone, ASUS A002), placed directly against the OSpRad's protective cover. Measurements were taken over multiple integration times in one-octave steps, ramped down from saturation point (typically ∼100 ms), to 1 ms (minimum value) and then back up to saturation point (ramping both ways to ensure the light source remained stable). Each unit's summed counts, *c*, across the spectral emission range of the light source were calculated as:
(2)

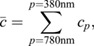
where *c* is the ADC response (count) at each photosite *p*, for sites between 380 and 780 nm (the approximate spectral emission range of the source). The expected linear count rate *r* is calculated from exposure time *t*:
(3)

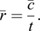
*r* is then scaled to max=1 for comparison between units. *r* was found to show a log-linear response to *c*, which could be modelled accurately with the function:
(4)

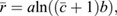
where *a* and *b* are coefficients fitted with least-squares regression, shown in Fig. S2. Fitting was performed in ImageJ (version 1.53). This function levels out as counts approach zero, which reduces noise compared with typical linearisation functions such as the Gamma variate, which also fits the linearisation function well, but causes considerable artificial noise as counts approach zero. The customised linearisation function subtends to zero with values below 1, whereas log-linear models become negative (functions shown in Fig. S2). Linearised counts at each photosite are then calculated as:
(5)

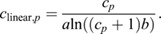
Any negative count values are assumed to follow the same function (the sign is switched, and switched back following linearisation) in order to avoid systematically amplifying any negative values.

#### Spectral sensitivity

**Fig. 2.**
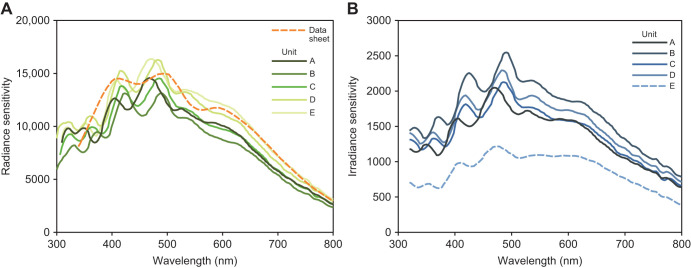
**Radiance (*S*_r_) and irradiance (*S*_i_) sensitivity functions for five OSpRad units.** (A) Radiance. (B) Irradiance. The orange dashed line shows the sensitivity published by the manufacturer (scaled to match the approximate sensitivity *y*-axis of the plot). Note that the lower irradiance sensitivity of unit E is due to a difference in its cosine-corrector construction (four layers of PTFE tape, whereas units A–D use a single 0.5 mm PTFE sheet sanded with 180 grit paper).

Spectral calibration should be performed using a light source with a known/measured emission spectrum. I attempted this with a deuterium source (Thorlabs SLS204); however, the large 650 nm spike reduced the effective dynamic range in order to avoid saturation. I also used a halogen source, but this was insufficient for calibrating the lower UV range and its extreme near infrared output appeared to cause second-order diffraction grating interference. I therefore used a standard xenon photography strobe flash (Neewer NW-14EXM) that had been converted to full spectrum by removing the plastic (UV-absorbing) protective covers and replacing them with diffuse 0.5 mm virgin PTFE sheet. The xenon source was used to illuminate a virgin PTFE sheet (20×20 cm, 1 mm thick) from behind to create a uniformly illuminated surface in a dark room. Radiance and irradiance were measured with a Jeti Specbos (1211UV) spectroradiometer with NIST-traceable calibration, ∼20 cm from the rear-illuminated surface. The Jeti unit is limited to <800 nm, so although C12880MA chips have sensitivity up to ∼880 nm, this was not measured. The OSpRad units were then used to measure the radiance and irradiance of the same surface from the nearest distance that did not cause saturation (∼100 cm away; this light source is strobed, so integration time cannot be used to limit exposure and the C12880MA chips have far higher sensitivity than the Specbos). This process allowed for calibration of the relative spectral sensitivity of the units, but given the differences in detection distance, could not give absolute values. Fig. S3 shows the radiance of the calibrated light source and linear (non-spectrally calibrated) OSpRad measurements of the same source. Absolute radiometric calibration was next performed using an AMOLED screen (as above, ASUS A002). The Specbos and OSpRad units were placed directly against the screen, which was displaying a white surface, for measurements of absolute intensity. Following calibration, radiance *L*_e_ (W sr^−1^ m^−2^ nm^−1^) is calculated at each photosite's peak wavelength (λ) as:
(6)

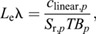
where *c*_linear,*p*_ is the average linearised count at each photosite *p*, *S*_r_ is radiance sensitivity, *T* is integration time (ms) and *B* is the spectral width of the bin (nanometres). Irradiance *E*_e_ (W m^−2^ nm^−1^) is calculated similarly, substituting *S*_r_ for *S*_i_ (irradiance sensitivity). The sensitivity functions, smoothed using a Gaussian filter (σ=3), are shown in Fig. 2. Note that these units are watts per nanometre, so to recover the energy of a given spectrum at each photosite, *L*_e_ or *E*_e_ must be multiplied by the bin width *B* at that photosite. Wavelength λ is calculated from *p* using a function with coefficients supplied by the manufacturer for each unit.

#### Cosine corrector

The cosine correctors were tested by illuminating each unit with a stable LED point light source (Phillips PC amber Luxeon) with a continuous current of 152 mA (∼2.7 V). The LED was attached to an arm that rotated around the OSpRad's cosine corrector with a radius of 660 mm. The OSpRad's surface was levelled with a spirit level, and the arm's angle was measured with an inclinometer. Irradiance was measured from angles of 0 deg (directly above the surface) to 80 deg and then back up to 0 deg, in 10 deg increments. Default measurement behaviour was used (auto-exposure, minimum of three scans). Irradiance was measured with the plastic protective covers removed to test optimal performance, and then again with the plastic covers in place. Results are shown in Fig. S4. The bare 0.5 mm PTFE cosine corrector performs close to the ideal cosine function down to roughly 70–80 deg, below which the housing casts a shadow over the surface. The PFTE tape-based cosine corrector has slightly poorer performance. Performance is reduced by the presence of the plastic cover in all cases (owing to specular reflection and refraction at acute angles, particularly below 50 deg). Nevertheless, performance is highly consistent among all four 0.5 mm PTFE cosine correctors.

#### Low-light performance

**Fig. 3.**
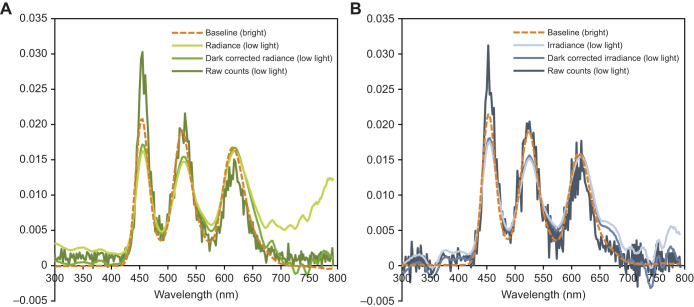
**Low-light performance testing for radiance and irradiance, showing the baseline measurements (taken under bright light conditions of 5.48 cd** **m^−2^ and 49.1** **lx, respectively), raw counts (*c*; see Materials and Methods), calibrated radiance or irradiance values, and dark-corrected values.** (A) Radiance in this example was 0.0013 cd m^−2^ (measured by averaging 80 scans of 30 s) and (B) irradiance was 0.0051 lx (average of 48 scans of 30 s). All metrics are scaled to area under the curve from 425 to 650 nm=1 for assessing relative spectral shape. All spectra shown were smoothed with a Gaussian function (sigma=3), with the exception of raw counts. Note that raw counts do not account for spectral sensitivity or linearisation.

Low-light performance was tested with an AMOLED screen (ASUS A002, as above), displaying a white square on a black surround, measured from 100 mm away. Screen brightness was reduced to its minimum, and the size of the square was reduced incrementally to assess the lowest light levels that produced usable spectral data. The three clearly defined emission peaks of the screen's LEDs are shown in Fig. 3. The level of acceptable noise will depend on the intended use of the resulting spectra, and can generally be reduced by averaging multiple exposures, and must ultimately be assessed by researchers. As such, I present unfiltered raw count data together with processed spectra for assessing low-light performance.

Irradiance measurements use the cosine corrector, which reduces sensitivity to low light. As such, radiance can be measured under lower light levels than irradiance. This testing used an OSpRad unit with a 0.5 mm sanded PTFE cosine corrector (unit B). Usable radiance spectra were measured down to 0.0013 cd m^−2^, and irradiance spectra were measured down to 0.0051 lx (see Fig. 3). Under these extreme low-light conditions there was an increase in estimated near-infrared energy caused by an underestimate of the dark value and subsequent amplification of noise in the low-spectral-sensitivity regions of the spectrum (infrared and UV). This is likely caused by scattered light inside the spectrometer, so cannot be accounted for by closing the shutter. To compensate for this effect, a dark correction was applied by subtracting 0.5 from raw radiance counts, and 0.2 from raw irradiance counts in the examples shown in Fig. 3.

## RESULTS AND DISCUSSION

### Calibration

Linearisation was accurately modelled by a function that had just two coefficients. The function was highly consistent among units, and model fit *R*^2^ values were between 0.989 and 0.998 (see Materials and Methods, Fig. S2). Spectral sensitivity was highly consistent above ∼500 nm; however, below that wavelength there are modest differences (see Fig. 2).

### Performance testing

Irradiance measurements require a cosine corrector that acts as a diffuser, collecting light from its outer surface and re-emitting it on the inner surface. Ideally, this should fit a Lambertian distribution with a cosine function. I tested two methods for constructing cosine correctors: one using multiple layers of easily sourced 0.1 mm PTFE (polytetrafluoroethylene) tape, and another using a sanded 0.5 mm thick virgin PTFE filter. Testing shows that the 0.5 mm thick filter had superior performance, nearly matching the cosine function (see Materials and Methods, Fig. S4). Low-light performance testing suggests the OSpRad system can record spectral radiance down to 0.001 cd m^−2^ and spectral irradiance down to 0.005 lx.

### Summary

OSpRad spectroradiometers based on Hamamatsu C12880MA chips offer a cost-effective solution for spectral radiance and irradiance measurements in the UV-A to near infrared range at low light levels. This makes them particularly well suited to behavioural and ecological measurements, and investigating the effects of ALAN, as demonstrated in our recent study investigating the effect of lighting on the landscape of fear in an endangered shorebird (Jolkkonen et al., 2023).

OSpRad units were able to measure spectral irradiance at around 0.005 lx with an acceptable level of noise (see Fig. 3), which is roughly between starlight and the lowest levels of moonlight (Hänel et al., 2018; Johnsen et al., 2006). Sensitivity was higher for radiance, measuring spectra at around 0.001 cd m^−2^, meaning it is able to measure spectra under the vast majority of typical night-time light intensities relevant to ALAN research; for example, the recommended luminance of street lights is 2 to 0.3 cd m^−2^, and the Milky Way is ∼0.0027 cd m^−2^ (Hänel et al., 2018). Nevertheless, higher sensitivity would be required in some circumstances such as under thick cloud cover or vegetation with no moonlight or ALAN. The system's UV sensitivity at around 66% relative to the peak is also well suited to measuring low-intensity UV light. Further work could assess whether low-light sensitivity can be improved through hardware or software solutions.

Spectral calibration of OSpRad units is a process that requires access to specialist, and expensive, equipment – such as a UV-visible light source and calibrated spectroradiometer – that could be a barrier to their widespread use. The data presented here from five units suggest that absolute sensitivity is comparatively uniform among units, whereas there are modest differences in spectral sensitivities, particularly below ∼500 nm. Therefore, if researchers require high levels of confidence in the accuracy of their recordings, each unit should be calibrated independently. However, if researchers were to use the data presented here for their otherwise uncalibrated units, the overall luminance/illuminance measurements produced would be comparatively accurate (particularly when considering the log-scale of light intensities that they will typically be used to measure). Exact measures of spectral intensity below ∼500 nm would be prone to error; however, the degree of error will be highly dependent on the shape of the spectra being measured and spectral sensitivities of the visual system. Careful experimental design may also be used to rule out major systemic bias as a result of imperfect unit-specific calibration. For example, uncalibrated OSpRad units could be used to reliably measure relative spectral shifts (e.g. proportions of UV to SW light) with high repeatability. The use of uncalibrated equipment is most problematic if colour measurements are being compared among multiple units with slight (unknown) differences in their spectral sensitivity. One possible solution to this would be to calibrate all OSpRad units against a single ‘master’ unit and accept some degree of absolute error, while maintaining cross-unit repeatability. Additionally, the spectral sensitivity functions (Fig. 2) appear to vary more in wavelength offset than overall shape, implying unit-specific calibration could be achieved by fitting the curves presented here to measurements of a spectrally consistent source (such as a halogen bulb), shifting a template sensitivity curve along until peaks are eliminated. Linearisation fitting shows that all units are behaving in a similar manner, with modest differences between units. For example, taking the two units with most different curves (units C and E), if each used the linearisation parameters of the other unit, the resulting *R*^2^ values would be 0.9984 (C fitted with E's parameters) or 0.9976 (E fitted with C's parameters). If users are not able to generate their own linearisation calibration, I recommend using unit C's parameters (*a*=0.14231, *b*=1.06125; see Fig. S2).

Cosine correctors that match a true cosine scattering performance are typically extremely expensive components constructed from materials such as Spectralon® sintered PTFE (Labsphere). For the OSpRad, I sought alternatives that could readily be used in low-cost or data-logging applications, where perfect cosine performance is typically not critical. Nevertheless, I demonstrated that a cosine corrector made from sanded 0.5 mm thick virgin PTFE sheet performed well in both transmission and scattering of light. Using a slightly thicker filter would likely improve cosine-related performance, while reducing low-light performance. The cosine corrector created from four layers of PTFE tape resulted in slightly poorer transmission and diffusion characteristics.

Future developments could investigate performance improvements such as high-light-intensity performance (sub-millisecond integration times will be required to measure sources >∼700 cd m^−2^), high dynamic range measurements, or use of a mechanical gimbal to generate hyperspectral images/spatial information. In releasing these open-source tools, I hope to facilitate research into monitoring and mitigating for the effects of ALAN.

## Supplementary Material

10.1242/jexbio.245416_sup1 Supplementary information
