# Supplementary material for: OSpRad: an open-source, low-cost, high-sensitivity spectroradiometer
Source: J Exp Biol. 2023 Jul 12;226(13):jeb245416. doi: 10.1242/jeb.245416 (PMC10357011; doi:10.1242/jeb.245416)
Supplement: Supplementary information [file jexbio-226-245416-s1.pdf]

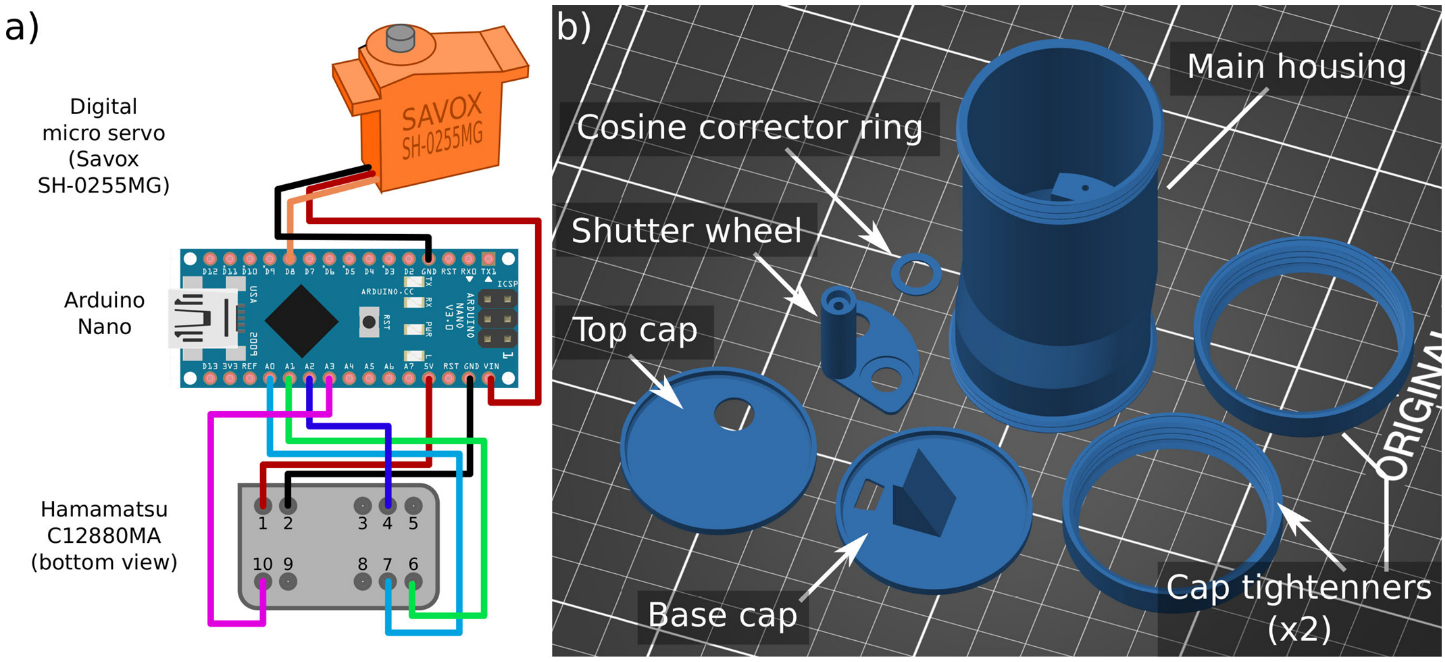

Fig. S1. Circuit diagram (a), showing connections between the microcontroller (Arduino Nano), spectrometer chip (C12880MA) and digital servo that controls the shutter/filter wheel. Note that the C12880MA chip and servo run from separate voltage rails, due to the voltage drop in the VIN's diode. b) shows 3D printed components required for construction.

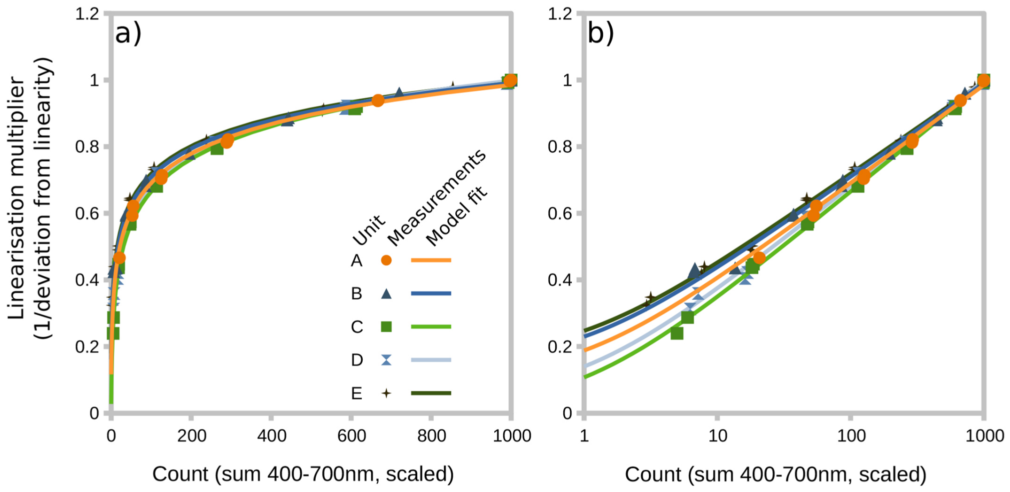

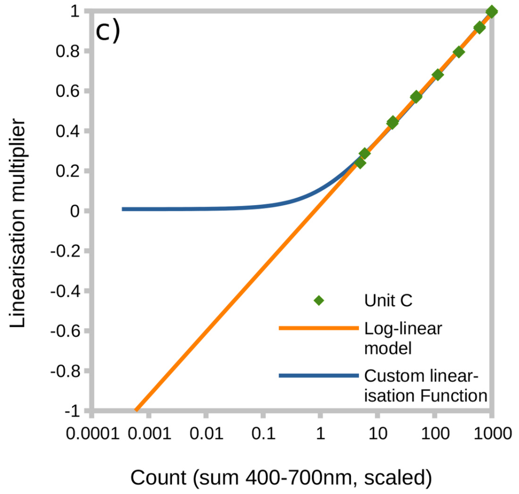

d)

Unit	*a*	*b*	R^2
A	0.12828	2.16327	0.9891
B	0.12249	3.258	0.9913
C	0.14231	1.06125	0.9984
D	0.13812	1.37537	0.9959
E	0.12032	3.89444	0.9976

Fig. S2. Linearisation modelling of five OSpRad units shown with linear a) and log (b) x-axes. X-axes show the sum of counts (*c*) from 400-700nm, of a stable light source measured at a range of integration times, then scaled to max=1000 (the saturation point at each pixel). The y-axis shows the linearisation multiplier *r* (count values are linearised by dividing them by this value, see text). c) shows the customised linearisation function and a log-linear function fitted to the sample data from unit C. Both models fit the observed data well, however the log-linear function creates large, negative values for small count data (<1), whereas the custom function subtends to zero. d) shows linearisation coefficients and model R^2 fits.

Journal of Experimental Biology • Supplementary information

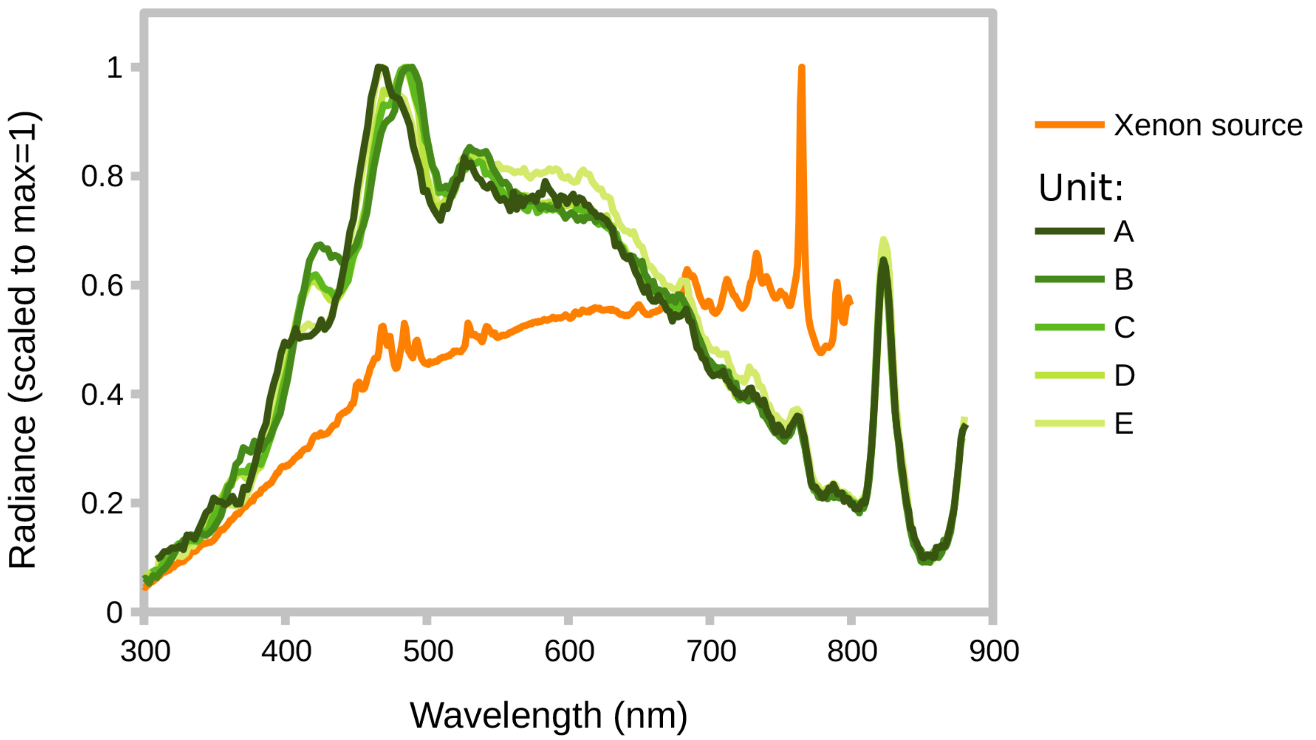

Fig. S3. Plots showing linearised radiance counts (c_{linear}) prior to spectral calibration from all 5 units (green) of the xenon light source used for spectral calibration. The calibrated radiance (as measured by a Jeti Specbos 1211UV) is shown in orange. All values have been scaled to max=1. Spectral sensitivity is calculated from the difference between the known radiance and measured radiance.

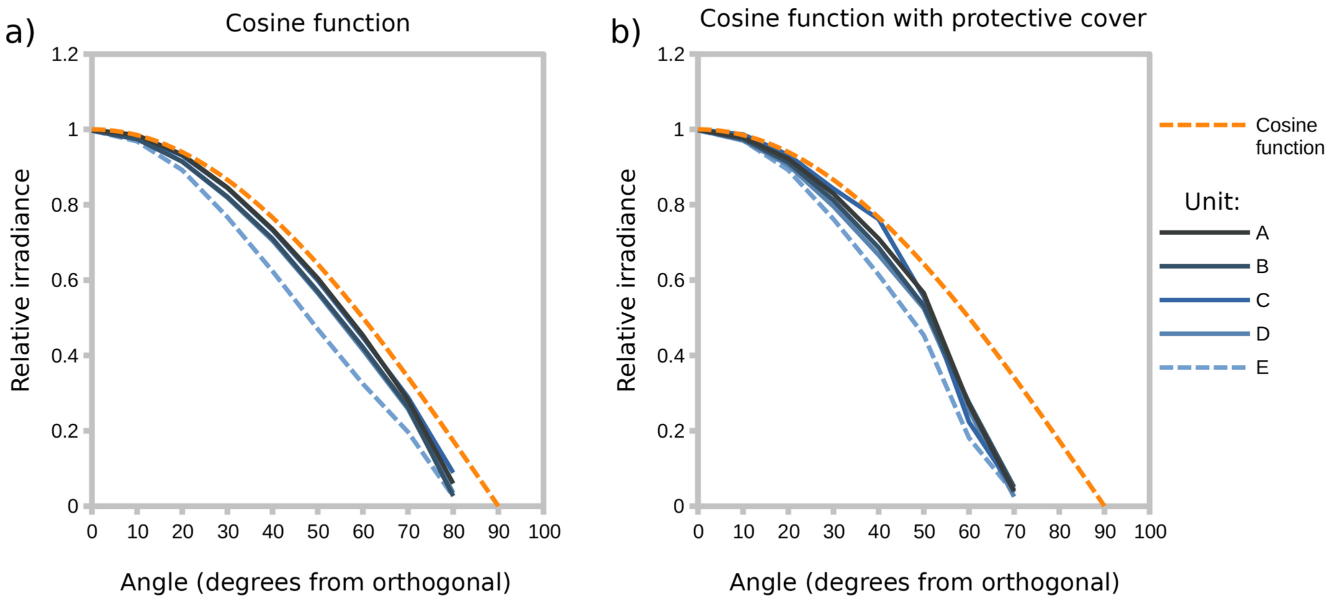

Fig. S4. Cosine corrector test data, with a bare surface (a) or plastic protective cover (b). An ideal cosine corrector would cause irradiance to fall off with the angle of illumination following the cosine function (shown in dashed orange). OSpRad units A-D use 0.5mm thick sanded PTFE filters, while E uses four layers of PTFE tape.
